# Quality-of-life survey of pancreatic cancer patients: a comparison between general public and physicians

**DOI:** 10.3389/frhs.2024.1275496

**Published:** 2024-07-12

**Authors:** Yuriko Sasahara, Yuki Takumoto, Kaname Watanabe, Hiroyuki Takeda, Kumiko Umemoto, Yu Sunakawa, Naoki Suzuki, Takashi Yoshioka, Satoshi Kobayashi, Makoto Ueno, Sho Nakamura, Manabu Akazawa, Hiroto Narimatsu

**Affiliations:** ^1^ Department of Medical Oncology, Yamagata Prefectural Central Hospital, Yamagata, Japan; ^2^ Department of Public Health and Epidemiology, Meiji Pharmaceutical University, Tokyo, Japan; ^3^ Cancer Prevention and Control Division, Kanagawa Cancer Center Research Institute, Yokohama, Japan; ^4^ Department of Genetic Medicine, Kanagawa Cancer Center, Yokohama, Japan; ^5^ Department of Clinical Oncology, St. Marianna University School of Medicine, Kawasaki, Japan; ^6^ Department of Clinical Oncology, Yamagata University, Yamagata, Japan; ^7^ Department of Gastroenterology, Kanagawa Cancer Center, Yokohama, Japan; ^8^ Graduate School of Health Innovation, Kanagawa University of Human Services, Kawasaki, Japan

**Keywords:** quality-of-life, pancreatic cancer, physicians, VAS, cTTO, EQ-5D

## Abstract

**Background:**

Quality-of-life (QOL) is important for cancer patients with poor prognosis. However, conducting a QOL survey with patients is difficult. Therefore, we conducted a QOL survey with physicians. Specifically, this study aimed to clarify how physicians assess QOL in patients with pancreatic cancer by conducting a survey and comparing the results between physicians and the general public.

**Methods:**

A survey was conducted by interviewing physicians administering chemotherapy to patients for recurrent/metastatic pancreatic cancer. This method is similar to that of the QOL survey conducted among the general public. Responses were evaluated using the composite time trade-off (cTTO) and the visual analog scale (VAS) for 11 pancreatic cancer status scenarios (survey scenarios). These scenarios consisted of patients’ health states as well as the types and grades of adverse events (AEs). Health status was classified into two categories: Stable disease (SD) and Progressive disease (PD). In addition, we conducted a survey using the EuroQol 5 Dimensions 5-Level (EQ-5D-5l) as reference values.

**Results:**

Twenty physicians responded to the survey. SD had the highest mean QOL value for both assessment methods (Physicians: 0.78, General public: 0.63), whereas PD had the lowest mean QOL value (Physicians: 0.15, General public: −0.12). The physicians assigned higher QOL values on both the VAS and cTTO than the general public did in all survey scenarios.

**Conclusions:**

The QOL values obtained from physicians were consistent with the degree of status in any assessment scenarios. Based on the differences in the QOL survey results between physicians and the general public, physicians tended to assign higher QOL values than the general public in cTTO and VAS assessments.

## 1 Introduction

Chemotherapy plays a major role in treating cancer with distant metastasis. In the 21st century, in addition to cell-killing drugs and molecularly targeted drugs, immune checkpoint inhibitors have emerged as anti-cancer drugs and have significantly contributed to prolonged survival (1). However, the cost of these drugs and overall medical expenses are increasing yearly, raising concerns about clinical and medical economic efficiency (2). In Japan, a medical economic assessment was introduced in 2019 as part of the medical technology assessment in the medical insurance system (3). The quality-adjusted life year (QALY) assessment is useful for medical economic assessments; however, evidence for the quality-of-life (QOL) value (utility value) required for calculation is limited (4).

Patient-reported outcome (PRO)/QOL assessment is one of the secondary endpoints in many recent phase III trials for various cancers, such as lung, esophageal, gastric, and breast cancers (5–8). The Food and Drug Administration (FDA) and the European Medicines Agency (EMA) issued guidance on the use of PRO/QOL and health-related QOL (HR-QOL) in 2009 and 2012, respectively, for cancer treatment assessment. In Japan, a PRO/QOL study group was established in January 2011 (9–11). Advances in chemotherapy are believed to emphasize the importance of prolonged survival and treatment selection based on QOL assessments.

Various questionnaires have been developed to assess HR-QOL. However, to calculate QALYs, one of the outcomes of cost-utility analysis, it is necessary to measure the utility value using preference-based measures (PBMs), including EuroQol five-dimensions (EQ-5D), Short Form six-dimensions (SF-6D), and the Health Utilities Index (HUI) (12–14). In particular, the EQ-5D questionnaire method of asking specific patients to provide their QOL values is widely and commonly used. In oncology, the QOL utility value measured by the EQ-5D is relatively stable for each cancer type, condition, and treatment (15). This method is recommended by NICE, as evidenced by its valuation using the time trade-off (TTO) method for the general public in the UK (16). Therefore, QOL is generally measured subjectively by the patient, and health technology assessment (HTA) organizations in other countries have emphasized economic assessment using the results of QOL assessment questionnaires, such as the EQ-5D. However, there are many instances of poor disease prognosis or an insufficient number of patients. Recently, the vignette-based method, which investigates QOL utility values using specific disease scenarios, has been widely used for the general public (17). In addition, Japan's cost-effectiveness assessment guideline (version 3) states that the QOL value calculation method using the TTO can be used as one of the measurement scales that can be converted to QALY, reflecting the value of the general public (18).

Pancreatic cancer, a typical cancer with a poor prognosis, is difficult to detect in its early stages and is often diagnosed as unresectable due to locally advanced or metastatic cancer. The treatment for unresectable pancreatic cancer is chemotherapy. There are several chemotherapy options with different possible adverse events (AEs) depending on the treatment choice. Depending on the severity of the side effects, QOL may be reduced. In pancreatic cancer patients with a poor prognosis, QOL is important, in addition to therapeutic efficacy. However, in QOL surveys concerning cancer patients, lack of data due to deterioration in patients’ health can be a problem. In a QOL survey of non-small cell lung cancer in older adults, the quantity of data decreased by 57%, and analysis results based only on data from patients who completed the survey did not reflect the overall QOL results and were biased for patients in good condition (19). The prognosis for pancreatic cancer is poorer than that for lung cancer, and the data are more likely to be insufficient. It is considered challenging based on the feasibility of collecting QOL data from clinical trials.

Therefore, we created pancreatic cancer scenarios and conducted a QOL survey among the general public in our previous study (20), believing that we could evaluate the impact of pancreatic cancer on QOL from the perspective of the general public. However, creating disease scenarios for pancreatic cancer, with its various complex and severe symptoms, has led to the suggestion that some disease scenarios are difficult for the general public to imagine and understand, as they may not have experienced such situations. Consequently, a proper assessment of the impact on QOL is limited. Therefore, by conducting a QOL survey using a recurrent or metastatic pancreatic cancer disease scenario and targeting physicians with a deeper understanding of the disease, we believe that the limitations of surveys targeting the general public could be examined. However, there are limitations in the assessment of QOL values based on disease scenarios for medical professionals, including physicians, due to reported discrepancies in the assessments of physicians and patients. For example, in the assessment of prostate cancer symptoms, peripheral neuropathy due to taxane preparations, and symptoms and side effects of anticancer drugs in the palliative area, physicians’ evaluation of QOL tends to be higher than that of patients, suggesting a discrepancy in patient assessments (21–24). The differences between physicians and patients are assumed to be largely due to their characteristics. Therefore, physicians are assumed to have a breaking point in a direction that is different from that of the general public. As such, we believe that comparing QOL survey results between physicians and the general public will help obtain an appropriate QOL value for recurrent/metastatic pancreatic cancer.

Assessing QOL in cancer is crucial, but challenges persist. First, although several questionnaires have been developed to assess QOL, conducting QOL surveys in cancer patients with poor prognoses, such as patients with pancreatic cancers, is difficult. Second, although QOL surveys for the public are increasingly gaining acceptance, appropriate responses are limited due to the characteristics of the disease. Third, QOL values obtained even for the same disease are known to vary depending on the culture, social conditions, education, etc., of each country. Therefore, the only studies that can be compared with the present study are QOL survey studies conducted on Japanese subjects, which are few and far between (25). These realities are frequently outlined in conventional QOL evaluation papers and are mirrored in subsequent vignette-based QOL survey studies of the general public for well-known cancers (26, 27). Therefore, we conducted a QOL survey of recurrent/distant metastatic pancreatic cancer scenarios with physicians and compared the results obtained with the QOL survey results previously obtained from the general public.

## 2 Materials and methods

### 2.1 Survey

A survey was conducted by interviewing (face-to-face interview) physicians (respondents) administering chemotherapy to patients for recurrent/metastatic pancreatic cancer. This method of surveying physicians is similar to that of the QOL survey conducted among the general public.

For the survey, we requested the cooperation of physicians who belong to the medical oncology or gastroenterology departments at three hospitals in Japan and who administer chemotherapy for recurrent/metastatic pancreatic cancer. The participants of the survey were physicians working at three hospitals as of February 2022. In the questionnaire, we examined the age, employment category, educational background, and marital status of the respondents.

Responses were evaluated using the composite time trade-off (cTTO) and the visual analog scale (VAS) for 11 pancreatic cancer status scenarios to be evaluated (survey scenarios). In addition, we conducted a survey using the EuroQol 5 Dimensions 5-Level (EQ-5D-5l) as reference values. The survey scenarios were created by utilizing those created in previous studies (20) and developed according to the recommendations in the ISPOR PRO Good Research Practice Task Force report (28, 29).

The cTTO compares two health statuses (“Full health” and “Suboptimal health state”) while changing the years of survival. It is a method of calculating the QOL value from the content of the responses when the two health statuses have the same value. The question is formatted such that patients are asked to select their preferred prognosis case, including worse than death (WTD), and the answers are highly reproducible, with the visual effects having a small impact. In addition, the QOL value can be calculated directly, which facilitates result interpretation. However, this format has a few limitations such as the following: (1) It has several questions that require time to answer, and trade-offs become difficult especially when there are many items to compare; (2) the framing effect affects the evaluability of highly serious diseases.

The EQ-5D is a method used to rate a specific health condition based on a 5-point scale for five items: degree of mobility, self-care, normal life, pain or discomfort, and anxiety or depression (12). This method has only a few items that are easy to answer and require less time. It has high comparability because it is used in various disease areas. However, due to its 5-item 5-point assessment, its responsiveness to detailed changes and symptoms without items is low. Since the minimum value of the conversion table for the Japanese population is −0.019, it does not correspond to lower values. In addition, the EQ-5D questionnaire was created to evaluate patients’ disease status. As such, the conversion table to the QOL value is based on the assessment results of the Japanese general public. Therefore, the conversion of the assessment results by physicians has not always been validated. The VAS is a method used to determine a specific health condition on a scale of 0–100 and calculate the QOL value (26). It is easy to visualize and answer, and it requires a short completion time. However, this method uses a scale; therefore, respondents tend to avoid choosing extremely positive or negative values, indicating that it is impossible to score worse health conditions, such as death.

The survey scenario was expressed with five items: “overview,” “physical symptoms (appetite, fatigue, and body pain),” “daily life at home and outside,” “mental state,” and “AEs” (Table 1). This scenario was created and used during the survey among the members of the general public. AE grades were defined by the Common Terminology Criteria for Adverse Events (CTCAE) versions 4 and 5 (30, 31).

**Table 1:** Definitions of health status scenario.

No	health status scenarios	Definition overview^a^
1	SD (Reference)	State in which symptoms are stable with chemotherapy. A state of no occurrence of adverse events associated with chemotherapy.
2	SD + Neutropenia G1/2	State of SD with grades 1–2 neutropenia.
3	SD + Neutropenia G3/4	SD with grades 3–4 neutropenia.
4	SD + FN	SD with grades 3–4 FN.
5	SD + Diarrhea G1/2	SD with grades 1–2 diarrhea.
6	SD + Diarrhea G3/4	SD with grades 3–4 diarrhea.
7	SD + Nausea/Vomiting G1/2	SD with grades 1–2 nausea and vomiting.
8	SD + Nausea/Vomiting G3/4	SD with grades 3–4 nausea and vomiting.
9	SD + Neuropathy G1/2	SD with grades 1–2 peripheral neuropathy.
10	SD + Neuropathy G3/4	SD with grades 3–4 peripheral neuropathy.
11	PD	The patient's condition is deteriorating despite chemotherapy treatment. Chemotherapy has already been stopped. Therefore, no adverse events are assumed Grades 3–4.)

SD, stable disease; FN, febrile neutropenia; PD, progressive disease; G, grade (adverse events).

^a^ These differ from the scenario used directly in the study.

The cTTO was answered in the same format as the computer-based response system used in QOL surveys of members of the general public (Supplementary Figure S1). The EQ-5D-5l and VAS were performed using the survey forms provided by EuroQOL (32, 33).

As for the actual survey procedure, after interviewing the respondents about their backgrounds, instructions on how to answer the questionnaires were explained in the following order: the EQ-5D-5l, VAS, and cTTO. Subsequently, respondents performed three cTTO exercises (evaluating ’suboptimal health state’ as wheelchair status, better than wheelchair status, and WTD status) and then answered 11 survey scenario questions with the cTTO. The three exercises were administered to help respondents grasp the concept of cTTO and understand how to answer the questions. These example questions are commonly used in cTTO assessments. After completing the exercises, the respondents randomly compared one of the 11 scenarios with the full health scenario. Specifically, they compared the scenario of surviving 10 years with that of living×years in full health and chose the situation they would prefer if they were in each scenario. The duration of survival in full health was adjusted by employing the ping-pong method, depending on the answers provided. This process was repeated until the respondents determined that their preferences for both scenarios were the same, and similar comparisons were made for all 11 scenarios. After completing the cTTO, all 11 survey scenarios were presented again, and they were rearranged starting from the scenario with the best health condition, as considered by the respondent. The respondents were then asked to answer the EQ-5D-5l and VAS in the new order.

This study was approved by the Institutional Review Board of Kanagawa Cancer Center (2021-Epidemiology-99), the Ethics Committee of St. Marianna University School of Medicine, and the Ethical Review Committee of Yamagata University Faculty of Medicine.

### 2.2 Statistical analysis

The primary and secondary endpoints were cTTO and QOL values, respectively, and they were derived from the EQ-5D-5l and VAS. QOL values were calculated from each physician's responses to the cTTO, EQ-5D-5l, and VAS, and summary statistics were calculated for each survey scenario.

The Welch t-test was performed to compare the cTTO and VAS survey results obtained from the general public with the current survey results obtained from physicians. Based on existing studies, the equivalence margin of 0.1 for minimal clinically important difference (MCID) was used for the analysis.

As this survey was targeted at members of the general public, the aggregated results based on the anonymized data were used as the control and compared with the survey results obtained from physicians.

Furthermore, to eliminate the impact of respondents who may not have a correct understanding of the cTTO concept or how to answer the questions, inconsistent responses to cTTO practice questions or identical answers to all questions in the actual survey were excluded. Specifically, the following were excluded: (1) Those who gave contradictory answers to cTTO exercises 1 and 2 (when the QOL value calculated in exercise 2 was higher than the QOL value calculated in exercise 1) or (2) those who answered all questions with a QOL value of −1, 0, or 1. Analyses were performed using SAS version 9.4 (SAS Institute, Cary, NC) and R software version 4.0.4 (34).

## 3 Results

**Figure 1:**
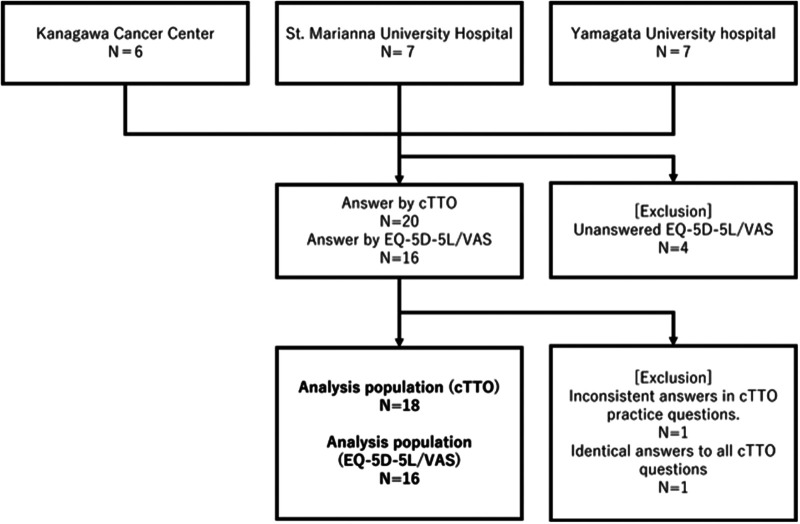
Participants’ flow diagram.

A questionnaire was sent to a total of 20 people. Responses were obtained from the 20 people for cTTO calculation; however, only 18 responses were included in the analysis. The remaining two were excluded because of inconsistencies. EQ-5D-5l or VAS responses were obtained from 16 respondents, all of whom were included in the analysis (Figure 1). The backgrounds of the physicians included in the analysis of the questionnaire survey are summarized in Table 2. Most respondents were male physicians in their 30s (age range: 26–62), and approximately 80% of them were married. They had similar educational backgrounds, such as university and graduate school graduates.

**Table 2:** Comparison of cTTO-derived QOL values between physicians and the general public.

Health status scenarios	Physician				General public				*P* value
	*N*	Mean	Std	Ranking	*N*	Mean	Std	Ranking	
SD (Reference)	18	0.78	0.21	1	201	0.63	0.02	2	0.013
SD + Neutropenia G1/2	18	0.76	0.21	2	201	0.65	0.03	1	0.058
SD + Neutropenia G3/4	18	0.76	0.19	3	105	0.51	0.04	4	0.000
SD + FN	18	0.47	0.41	10	201	0.32	0.04	8	0.163
SD + Diarrhea G1/2	18	0.70	0.27	5	105	0.50	0.04	5	0.012
SD + Diarrhea G3/4	18	0.55	0.29	8	201	0.31	0.04	9	0.005
SD + Nausea/Vomiting G1/2	18	0.62	0.25	6	201	0.42	0.04	6	0.006
SD + Nausea/Vomiting G3/4	18	0.48	0.25	9	105	0.24	0.06	10	0.005
SD + Neuropathy G1/2	18	0.72	0.21	4	105	0.54	0.04	3	0.008
SD + Neuropathy G3/4	18	0.62	0.28	6	201	0.37	0.04	7	0.002
PD	18	0.15	0.52	11	201	−0.12	0.04	11	0.052

SD, stable disease; FN, febrile neutropenia; PD, progressive disease; G, grade (adverse events).

**Figure 2:**
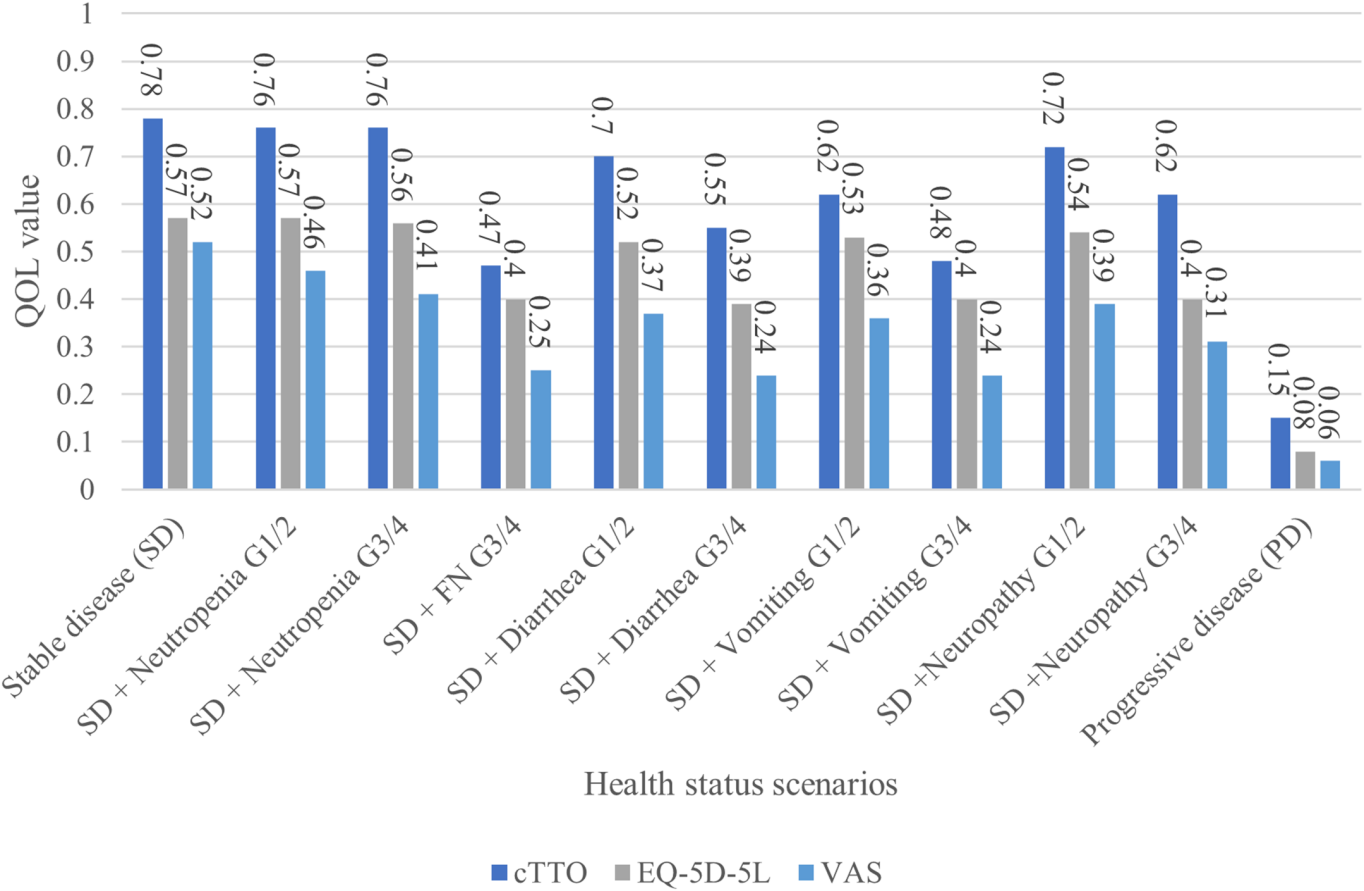
Comparison of the physicians’ assessment results.

Figure 2 shows the QOL values for cTTO, EQ-5D, and VAS survey results of the physicians for each scenario. Stable disease (SD) had the highest QOL value for both assessment methods (cTTO: 0.78, EQ-5D-5l: 0.57, VAS: 0.52), whereas progressive disease (PD) had the lowest QOL value (cTTO: 0.15, EQ-5D-5l: 0.08, VAS: 0.06). In each scenario, the values were in the order of cTTO>EQ-5D-5l>VAS.

Regarding cTTO, the physicians assigned higher QOL values than those assigned by the general public in all survey scenarios. (Table 3) Regarding the order of QOL values in the survey scenarios, although the differences between the respondents were not significant, the physicians ranked febrile neutropenia (FN) grades 3–4 (G3/4) lower than diarrhea G3/4 and nausea/vomiting G3/4 (physicians: 10th, general public: 8th). The rankings of G3/4 neutropenia and grades 1–2 (G1/2) peripheral neuropathy were reversed between physicians and the general public.

**Table 3:** Comparison of VAS-derived QOL values between physician and the general public.

Health status scenarios	Physician				General public			
	*N*	Mean	Std	Ranking	*N*	Mean	Std	Ranking
SD (Reference)	16	0.52	0.20	1	105	0.47	0.019	1
SD + Neutropenia G1/2	16	0.46	0.20	2	105	0.44	0.019	2
SD + Neutropenia G3/4	16	0.41	0.19	3	105	0.35	0.019	3
SD + FN	16	0.25	0.16	8	105	0.20	0.017	8
SD + Diarrhea G1/2	16	0.37	0.20	5	105	0.30	0.018	6
SD + Diarrhea G3/4	16	0.24	0.17	9	105	0.19	0.017	10
SD + Nausea/Vomiting G1/2	16	0.36	0.18	6	105	0.30	0.018	5
SD + Nausea/Vomiting G3/4	16	0.24	0.15	9	105	0.19	0.016	9
SD + Neuropathy G1/2	16	0.39	0.18	4	105	0.33	0.019	4
SD + Neuropathy G3/4	16	0.31	0.17	7	105	0.25	0.018	7
PD	16	0.06	0.22	11	105	0.11	0.020	11

SD, stable disease; FN, febrile neutropenia; PD, progressive disease; G, grade (adverse events).

By comparing the relative values of cTTO using SD as a control, no difference was observed in the degree of decline in QOL values for FNG3/4 between the physicians and the general public (Supplementary Table S1). Only neutropenia G1/2 scored higher in the general public. There was almost no inter-group difference in diarrhea and peripheral neuropathy for G1/2; however, for G3/4, the values assigned by the general public were smaller than those assigned by the physicians by approximately −0.1. The respondents from the general public scored neutropenia G3/4, nausea/vomiting G1/2, and G3/4 as low as approximately −0.05.

Physicians tended to assign higher QOL values on the VAS than the general public, similar to the cTTO (Table). The rankings of the survey scenarios showed no significant difference between the respondents.

Regarding the relative values of VAS using SD as the control, the difference in the degree of decline in QOL between the physicians and the general public was not significant in any scenario, except for PD. (Supplementary Table S2). The respondents from the general public assigned higher scores to neutropenia G1/2 and PD.

Relative to the SD + no AEs control, the study scenario with the least variation in QOL values was neutropenia G1/2, followed by neutropenia G3/4. The study scenarios with the highest variation in QOL values from SD, excluding PD, were febrile neutropenia G3/4 (−0.31) for cTTO, diarrhea G3/4 (−0.18) for EQ-5D, and nausea and vomiting G3/4 (−0.27), in addition to the two aforementioned scenarios for the VAS. Disutility due to AEs was greater with the cTTO and VAS than with the EQ-5D. (Supplementary Figure S2).

## 4 Discussion

In this study, we analyzed the QOL values assigned by physicians for recurrent or distant metastatic pancreatic cancer. First, the QOL values obtained were consistent with the status of the assessment scenarios, regardless of whether the assessment method was cTTO or EQ-5D-5l. Second, SD had the highest QOL value for both assessment methods (cTTO: 0.78, EQ-5D-5l: 0.57, and VAS: 0.52), whereas PD had the lowest QOL value (cTTO: 0.15, EQ-5D-5l: 0.08, and VAS: 0.06). In each scenario, the values were in the order of cTTO>EQ-5D-5l>VAS. This indicates that the QOL value of SD, which was the best status, was the highest, and the QOL value of PD was the lowest in all assessments. AEs were ranked between SD and PD in terms of QOL, and disutility increased according to the grade. In addition, only two respondents with low engagement gave inconsistent responses to the exercises or assigned the same QOL value to all the questions. Based on these findings, the respondents of this study could appropriately evaluate the scenarios’ situations and their impact on QOL; therefore, the obtained responses were considered consistent.

A comparison of the relative QOL values derived from the cTTO and VAS calculated using SD + no AEs as the control showed that neutropenia had a smaller degree of disutility than other adverse reactions, regardless of the severity (Supplementary Figure S2). The results suggested that the effects of neutropenia are limited to laboratory values, and its impact on patients’ physical functions is minimal. There was a difference of ≥0.1 for other Grade 3/4 AEs, regardless of the assessment method. In general, a clinically meaningful change in the QOL value of the EQ-5D in cancer is considered to be approximately 0.07–0.12 (35). Therefore, Grade 3/4 AEs are considered to have a large clinical impact. Neutropenia, even at Grade 3/4, is an AE assessed through blood sampling and generally does not appear as a subjective symptom in patients. Febrile neutropenia presents with at least subjective symptoms of fever. In addition, other AEs such as nausea and diarrhea are perceived by patients as symptoms. Based on their daily clinical experience, physicians are aware that AEs perceived by patients are side effects that cause physical pain; therefore, we believe that they considered these events to have a significant effect on patients’ QOL. Furthermore, a retrospective study on the relationship between side effects and the QOL of patients with advanced pancreatic cancer reported that anorexia, pain, and peripheral neuropathy were significantly associated with decreased QOL (36). Based on this study, side effects perceived by patients as subjective symptoms can be inferred to have a significant impact on QOL.

Moreover, between the three assessment methods, the cTTO had a higher QOL value than the EQ-5D and VAS (Figure 2). As the cTTO, which directly measures utility, is time-consuming and difficult to measure with actual patients, many studies targeting cancer patients have used the EQ-5D. The EQ-5D can convert the value measured by the patient into a value measured by the cTTO, and a conversion table (tariff) for Japan is available. As measuring the TTO in cancer is challenging, comparing whether other cancer types show similar trends is also challenging. The QOL value was higher in the cTTO than in the EQ-5D because the EQ-5D directly evaluates physical function (degree of mobility, personal care, and daily life), pain, and anxiety. Therefore, even if the status was SD, the EQ-5D may have reflected the disease state of metastatic pancreatic cancer, which is more severe than the normal state. Regarding why the cTTO>VAS, the VAS ceiling effect skews the score toward the higher side in good conditions, and the floor effect skews the score toward the lower side in poor conditions. The disease characteristics of metastatic pancreatic cancer are believed to have had a stronger effect. The cTTO>EQ-5D in the comparison of relative values probably due to the evaluation of the EQ-5D-5l on a 5-point scale; therefore, the effect of aggravation of AEs on the value was small. In the cTTO, disutility in AE G3/4 was 0.16–0.31, which was considerably higher than the minimal clinically important difference (MCID) (0.07–0.12) (Supplementary Figure S2). The cTTO can be used to directly calculate the QOL value; however, whether the QOL value of AE G3/4 based on the cTTO in this survey, where disutility deviated from the MCID, is appropriate as a utility value must be carefully determined.

By comparing the cTTO and VAS assessments between the physicians and the general public, we observed that the general public and physicians have different perceptions of pancreatic cancer pathology. In both assessments, physicians tended to assign higher QOL values than those assigned by the general public. Previous studies have confirmed that the general public tends to assign low QOL values. A previous study that investigated the impact of low back pain on QOL values in patients and the general public showed that the QOL values for acute low back pain were lower by 0.098 points (95% CI: 0.082–0.015) in the general public than in patients (37). Patients with a specific disease have a higher degree of understanding and adaptability to their condition than the general public, suggesting that the two groups do not necessarily have the same QOL values. In this study, metastatic pancreatic cancer had a poorer prognosis than other types of cancer, and the expected survival time for this type of cancer is short. Therefore, we believe that the general public assumed a considerably worse condition than the disease scenario of metastatic pancreatic cancer. However, the results suggested that physicians, who have clinical experience with various pancreatic cancer patients, assigned higher QOL values than the general public, even when physical functions were worsened. In addition, there were differences in the assessment order of QOL values in the survey scenarios. This may be due to the difficulty faced by the general public in imagining AEs such as neutropenia and FN, whereas it was easy to imagine diarrhea, nausea, and vomiting based on patients’ experiences. Therefore, we believe that physicians evaluated neutropenia and FN as having a smaller and greater impact, respectively, on QOL than the general public. In terms of relative values, the general public rated FN as having the same degree of disutility as the physicians; however, diarrhea and nausea/vomiting were rated as having more disutility. From these results, we believe that the ease of imagining AEs affected the QOL value. Conversely, the general public may evaluate an AE such as FN that is difficult to imagine at the same degree of disutility as an oncologist's assessment; therefore, we believe that the responses of the general public and physicians to the survey scenarios are reproducible and that the validity of the survey scenarios does not pose a problem. Compared with the general public in terms of relative values of the VAS, the differences between groups in each survey scenario were almost negligible. However, we believe that the QOL values were lower than those of the cTTO because the QOL values tended to cluster around smaller values due to the ceiling effect. This suggests that the difference in severe side effects may have been underestimated (Supplementary Table S2).

There was no significant difference in the QOL score ranking due to differences in the cTTO and VAS assessments. Contrarily, although the QOL value of each assessment scenario deteriorated due to the ceiling effect in the VAS, the difference in the relative value of disutility due to AEs against SD was similar between the assessment groups. In the assessment of PD, physicians assigned a score of 0 or higher, regardless of the assessment method, and did not result in WTD as in the general public. The physical condition for PD in metastatic pancreatic cancer is assumed to be highly serious and difficult for ordinary people to imagine. However, based on the assessment results obtained from oncologists, the value fluctuates between 0.06 and 0.15 depending on the assessment method, suggesting that the situation is not necessarily WTD. When conducting a QOL assessment using the cTTO in a serious disease scenario, although the assessment is usually assumed to skew to −1 or 0 (framing effect), in this study, the impact of the framing effect may have been mitigated by the familiarity of respondents (physicians) with the actual situation of pancreatic cancer (38). From the above, the QOL assessment using the target disease scenarios is different between the general public and physicians. By evaluating the QOL value of a highly severe disease status such as metastatic pancreatic cancer, it became clear that the QOL value cannot always be assessed appropriately in a survey among the general public. In particular, it was suggested that conditions associated with physical and mental disabilities such as PD may be difficult to imagine.

### 4.1 Limitations

This study had some limitations. First, the sample size of the targeted physicians was small. The study aimed to conduct exploratory descriptive research; therefore, the required sample size was not specified. Furthermore, since the physicians who participated in this study are engaged in the daily medical care of pancreatic cancer patients, it is easier for them to imagine pancreatic cancer patients who match the details of the health status scenarios provided in the assessment, and we assume that the extent of their impact on QOL will be shared. In addition, respondent engagement was extremely high. Only two of the respondents were excluded from the survey due to engagement issues. Although the sample size was not large, the order of summary statistics of QOL values in each health status scenario resulted in interpretable results.

Second, the survey scenario conditions were assumed to represent the clinically worst-case scenarios. The survey scenarios used in this study were created according to the existing creation process frequently used for the cTTO. However, in the cTTO, questions were asked based on the assumption that each disease state will continue for 1 year; therefore, it can be imagined that the worst state of each disease will continue for 1 year. Furthermore, in clinical practice, symptomatic therapy is implemented, and anticancer drugs are reduced or discontinued depending on AEs and worsening medical conditions. Therefore, the presented disease state does not necessarily last for a long time. These findings suggest that the QOL value calculated for each disease state may be underestimated compared to that in actual clinical practice.

Third, this study analyzed physician-evaluated QOL values for recurrent or metastatic pancreatic cancer. In addition, the discrepancy between the QOL survey results of physicians and the general public suggested that the QOL value assigned by the general public for metastatic pancreatic cancer may not always be evaluated appropriately. In particular, the disutility in QOL value derived from the cTTO assessments of the general public was greater than the QOL values assigned by physicians, and the disutility in severe AEs and conditions was significantly greater than the disutility and MCID observed in existing studies. Therefore, it is necessary to consider the QOL value derived from the general public's cTTO assessments as representative of the QOL value of distant metastatic pancreatic cancer, especially considering its similarity to the cTTO values among physicians. From the perspective of disutility due to AEs, the results of the EQ-5D-5l obtained from physicians were suggested to be the most explainable QOL values. From the results of this study, determining whether the QOL values assigned by the general public or those assigned by the physicians are closer to patients’ QOL values is difficult. Considering the discrepancy in values between the general public and physicians, the QOL values likely also differ between physicians and patients, since the physicians in this study tended to assign higher QOL values than did patients. In general, QoL values have different preferences depending on the characteristics of the respondent and should be selected according to the evaluation perspective (39). For example, when conducting cost-effectiveness analyses from a social perspective, preference should basically be given to QoL values based on the patients or the general public. If the analysis is from the healthcare provider's perspective, we could consider the physician's preference (40). However, in diseases such as metastatic pancreatic cancer, where various disease states occur in a complex and severe manner, our findings suggest that assessing the survey results among physicians and the general public may aid the appropriate assessment of QOL values in pancreatic cancer. This may mean that, although it is important to select the population for which QoL values should be measured according to the assessor's perspective as a reference for policy decisions, depending on the disease status, QoL values derived from a single population may not be used directly for cost-effectiveness analysis.

In addition, PD showed an exceedingly small QOL value of 0.08, even in the EQ-5D-5l, and the difference of 0.15 in the cTTO was unclear. In the future, as an effect of extremely low QOL value for PD, we believe further investigation is necessary to determine whether this reflects the disease-specific condition of metastatic pancreatic cancer with a poor prognosis and whether QOL values are valid if this reflects a serious condition.

## 5 Conclusion

We analyzed the QOL values of recurrent or metastatic pancreatic cancer evaluated by physicians treating pancreatic cancer. In the assessment of disease scenarios that are difficult for the general public to imagine, such as highly severe medical conditions, the results suggested that conducting a survey among physicians may help in the assessment of appropriate QOL values.

## Data Availability

The datasets presented in this article are not readily available because data cannot be shared publicly due to ethical restrictions. Requests to access the datasets should be directed to data described in the manuscript will be made available upon application (contact via the corresponding author) for researchers who meet the criteria for data sharing.
