# Supplementary material for: Quality-of-life survey of pancreatic cancer patients: a comparison between general public and physicians
Source: Front Health Serv. 2024 Jul 12;4:1275496. doi: 10.3389/frhs.2024.1275496 (PMC11272646; doi:10.3389/frhs.2024.1275496)
Supplement: Supplementary file 1 [file Datasheet1.docx]

Supplementary Material

**Supplemental Table 1. Comparison of cTTO-derived relative QOL values in physician and general populations（Reference scenario: SD）**

| Health status scenarios | Physician | | | General population | | | Difference |
| --- | --- | --- | --- | --- | --- | --- | --- |
|  | N | Mean | Std | N | Mean | Std |  |
| SD (Reference) | 18 | - | - | 201 | - | - | - |
| SD + Neutropenia G1/2 | 18 | -0.02 | 0.08 | 201 | 0.02 | 0.27 | 0.03 |
| SD + Neutropenia G3/4 | 18 | -0.03 | 0.07 | 105 | -0.09 | 0.32 | -0.06 |
| SD + FN | 18 | -0.31 | 0.39 | 201 | -0.31 | 0.39 | 0.00 |
| SD + Diarrhea G1/2 | 18 | -0.08 | 0.16 | 105 | -0.10 | 0.27 | -0.02 |
| SD + Diarrhea G3/4 | 18 | -0.23 | 0.18 | 201 | -0.33 | 0.42 | -0.10 |
| SD + Nausea/Vomiting G1/2 | 18 | -0.16 | 0.14 | 201 | -0.21 | 0.38 | -0.05 |
| SD + Nausea/Vomiting G3/4 | 18 | -0.30 | 0.21 | 105 | -0.36 | 0.43 | -0.06 |
| SD +Neuropathy G1/2 | 18 | -0.06 | 0.18 | 105 | -0.06 | 0.29 | 0.00 |
| SD +Neuropathy G3/4 | 18 | -0.16 | 0.26 | 201 | -0.26 | 0.41 | -0.11 |
| PD | 18 | -0.63 | 0.46 | 201 | -0.75 | 0.52 | -0.12 |

SD: stable disease; FN: febrile neutropenia; PD: progressive disease; G: grade (adverse events)

**Supplemental Table 2. Comparison of VAS-derived relative QOL values in physician and general populations（Reference scenario: SD）**

| Health status scenarios | Physician | | | General population | | | Difference |
| --- | --- | --- | --- | --- | --- | --- | --- |
|  | N | Mean | Std | N | Mean | Std |  |
| SD (Reference) | 18 | - | - | 201 | - | - | - |
| SD + Neutropenia G1/2 | 18 | -0.06 | 0.11 | 105 | -0.03 | 0.13 | 0.03 |
| SD + Neutropenia G3/4 | 18 | -0.10 | 0.09 | 105 | -0.12 | 0.14 | -0.02 |
| SD + FN | 18 | -0.26 | 0.12 | 105 | -0.27 | 0.20 | -0.01 |
| SD + Diarrhea G1/2 | 18 | -0.14 | 0.08 | 105 | -0.17 | 0.18 | -0.03 |
| SD + Diarrhea G3/4 | 18 | -0.26 | 0.08 | 105 | -0.28 | 0.18 | -0.02 |
| SD + Nausea/Vomiting G1/2 | 18 | -0.15 | 0.06 | 105 | -0.17 | 0.17 | -0.02 |
| SD + Nausea/Vomiting G3/4 | 18 | -0.27 | 0.07 | 105 | -0.28 | 0.16 | -0.01 |
| SD +Neuropathy G1/2 | 18 | -0.12 | 0.10 | 105 | -0.14 | 0.14 | -0.01 |
| SD +Neuropathy G3/4 | 18 | -0.20 | 0.10 | 105 | -0.22 | 0.17 | -0.02 |
| PD | 18 | -0.43 | 0.19 | 105 | -0.36 | 0.22 | 0.07 |

SD: stable disease; FN: febrile neutropenia; PD: progressive disease; G: grade (adverse events)

**Supplemental Figure 1. Example of a display screen in a composite time trade-off (English translation)**

**Supplemental Figure 2. Comparison of relative QOL values as physician assessment result（Reference scenario: SD）**


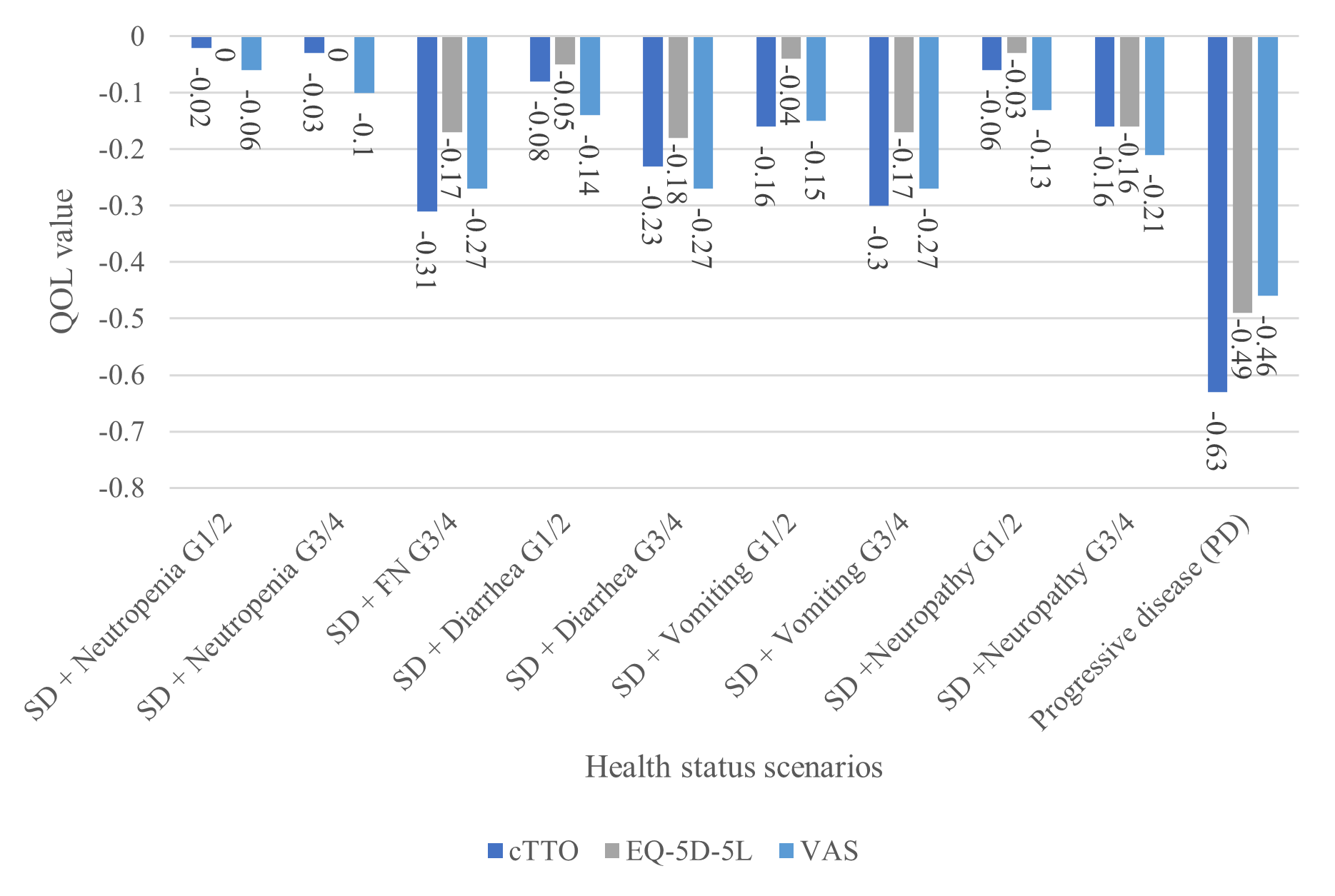


SD: stable disease; FN: febrile neutropenia; PD: progressive disease; G: grade (adverse events)
